# Supplementary material for: An Exogenous Ketone Ester Slows Tumor Progression in Murine Breast and Renal Cancer Models
Source: Cancers (Basel). 2024 Oct 4;16(19):3390. doi: 10.3390/cancers16193390 (PMC11476193; doi:10.3390/cancers16193390)
Supplement: Supplementary file 1 [file cancers-16-03390-s001.zip › cancers-3213364-supplementary.pdf]

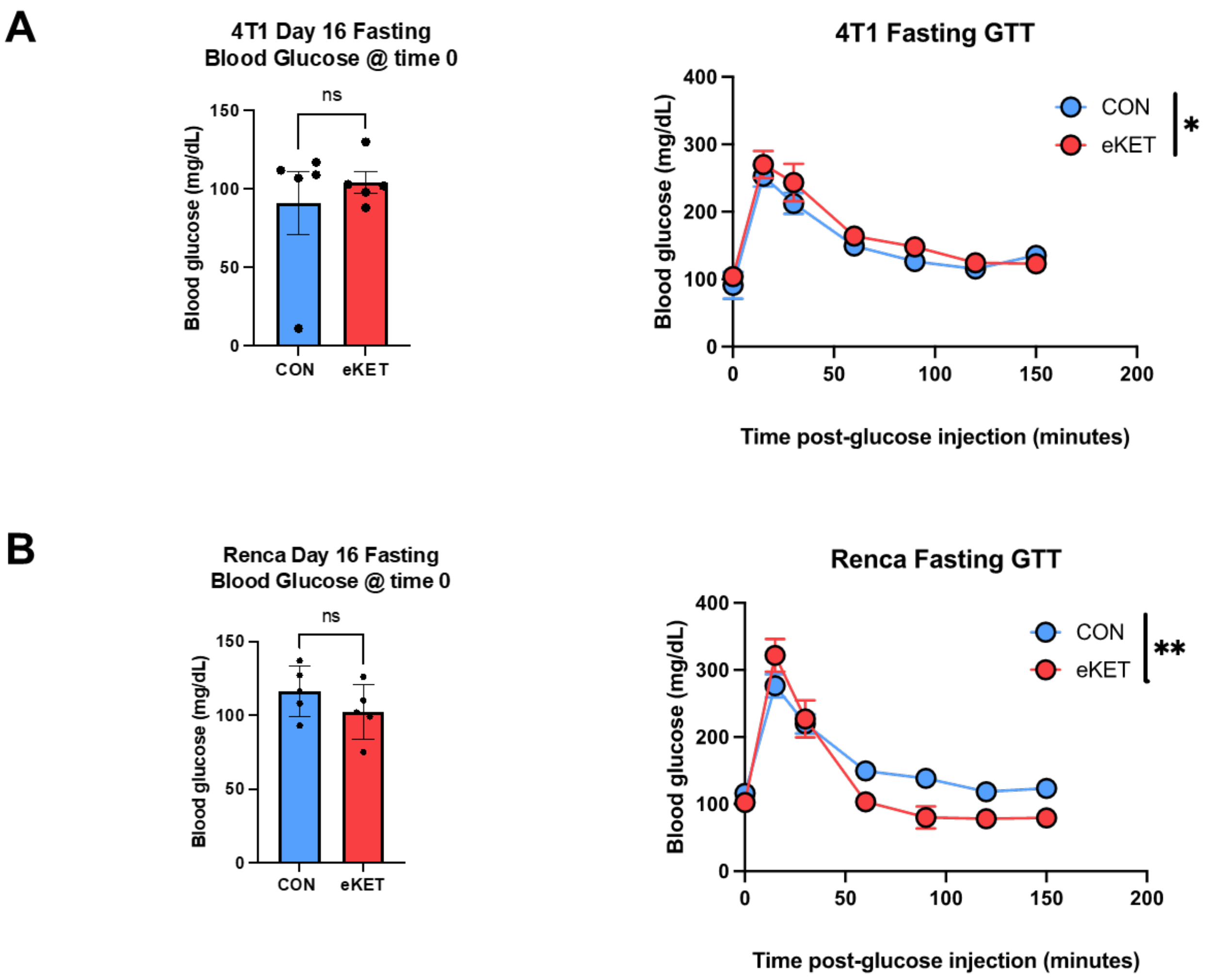

Figure S1. Supplemental Figure 1. Fasting blood glucose and glucose tolerance testing (GTT) in 4T1-Luc **(A)** and Renca-Luc **(B)** tumor-bearing mice shows exacerbated glucose clearance in mice with renal tumors on the eKET diet. At day 16 post-challenge, mice were fasted overnight and injected intraperitoneally with glucose (@ 2g/kg). Blood glucose was measured at the indicated time points after injection. Data are presented as mean ± SEM. **p* <0.05, ***p* <0.01 for eKET vs CON by two-way ANOVA with Tukey's multiple comparison test. CON, control; eKET, exogenous ketone ester.

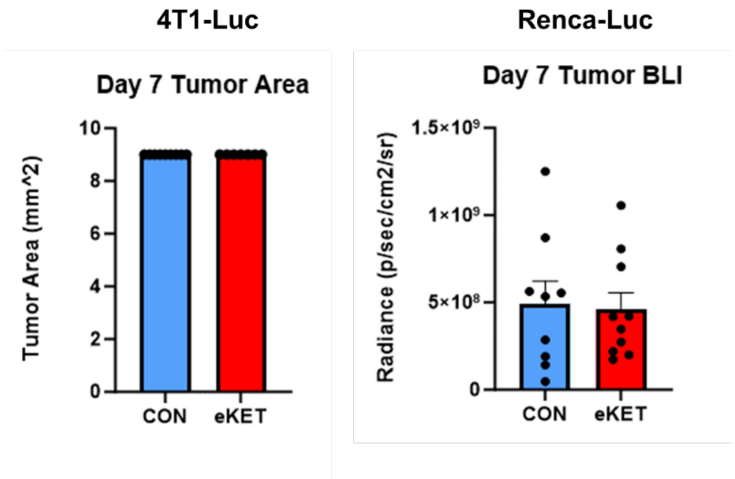

Figure S2. The exogenous ketone ester (eKET) diet does not alter early tumor growth. Mice were implanted with 4T1-Luc or Renca-Luc tumors and provided either control (CON) or eKET-supplemented diets. Tumor burdens were measured by venier calipers or BLI for 4T1-Luc and Renca-Luc respectively at day 7 post-tumor implantation. Data are presented as mean ± SEM. Statistical significance was determined using either parametric t-tests or non-parametric Mann-Whitney U tests, depending on the data distribution.

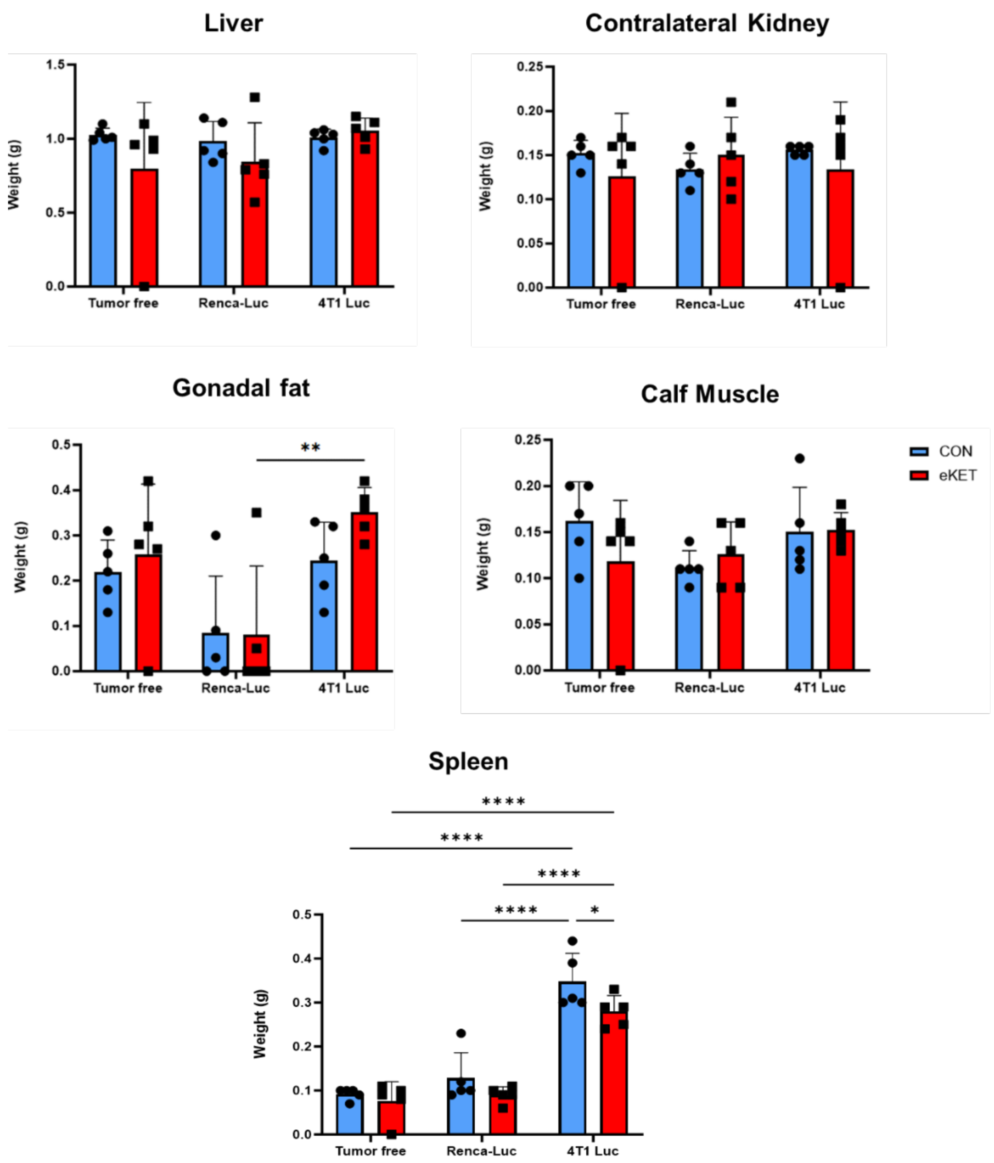

Figure S3. Effects of exogenous ketone ester (eKET) supplementation on organ and muscle weights in tumor-free versus tumor-bearing mice. Mice were implanted with 4T1-Luc or Renca-Luc tumors and provided either control (CON) or eKET-supplemented diets. Tumor-free mice were started on diets at the same time. Organ and muscle weights were measured at study endpoint (Day 21 for tumor-bearing mice). Data are presented as mean ± SEM. Statistical analysis was performed by two-way ANOVA with Sidak's multiple comparisons test (liver, contralateral kidney, gonadal fat, calf muscle, spleen) * $p < 0.05$, ** $p < 0.01$, **** $p < 0.0001$ for indicated comparisons.

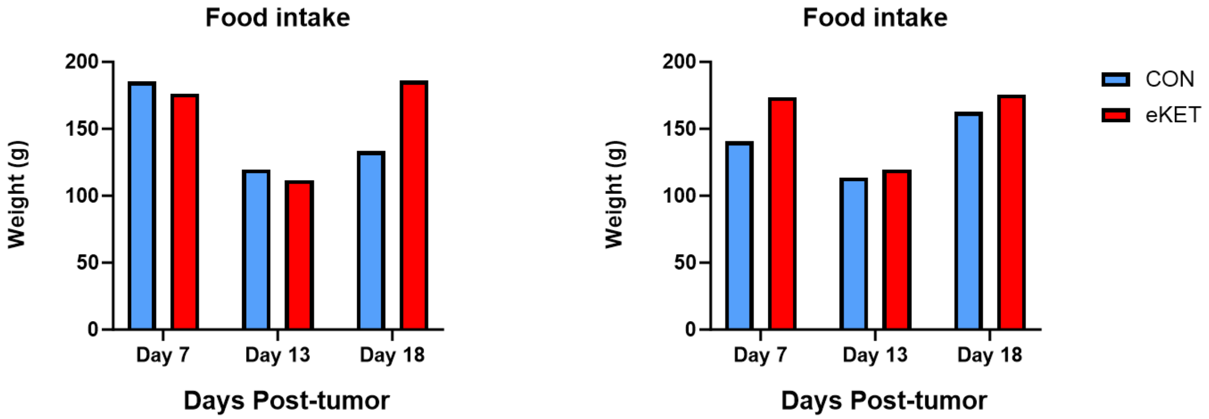

Figure S4. Food intake in exogenous ketone ester (eKET) and Control (CON) diet mice with 4T1-Luc and Renca-Luc tumors at Day 7, 13 and Day 18 post-tumor challenge. Mice were implanted with 4T1-LUC or Renca-LUC tumors and provided either control (CON) or eKET-supplemented diets. Food intake by cage was measured on the indicated days post-tumor implantation.

Table S1. List of intratumoral DEGs from mice with 4T1-Luc tumors (eKET vs Control)

**Downregulated**				
**Gene**	**log2FoldChange**	**Fold Change**	***p*-value**	***p*-adj value**
Aqp9	-3.6877	-12.8857	2.23E-06	0.001658
Tlr5	-2.87324	-7.32709	4.60E-06	0.001714
Cd40	-1.3411	-2.53344	2.14E-05	0.005307
Wnt4	-4.60627	-24.3571	3.26E-05	0.006071
Gli1	-4.14893	-17.7399	4.63E-05	0.006905
Ptger4	-1.12187	-2.17629	0.000132	0.0126
Fgfr1	-2.02733	-4.0765	0.000136	0.0126
Zeb1	-1.10592	-44.3333	0.000189	0.0126
Ncam1	-5.47032	-2.15236	0.000189	0.0126
Cdkn2b	-2.34091	-5.06622	0.000208	0.0126
Blk	-5.70834	-52.2855	0.00022	0.0126
Ttc30a1	-3.81876	-14.1111	0.000373	0.019834
Slc16a1	-4.48864	-22.4499	0.000419	0.020835
Wnt10a	-2.09433	-4.27028	0.000487	0.022661
Ccr4	-4.79874	-27.8333	0.000565	0.024781
Ccl20	-3.85536	-14.4737	0.000755	0.03124
Hras	-3.21501	-9.2857	0.000917	0.035971
Shc2	-1.97314	-3.92622	0.000988	0.036812
H2-pa	-5.6363	-49.7388	0.001157	0.041041
Magea4	-4.58033	-23.9231	0.001219	0.041283
Jup	-3.02106	-8.11764	0.001398	0.045279
Edn1	-1.80143	-3.48566	0.001506	0.046737
**Upregulated**				
**Gene**	**log2FoldChange**	**Fold Change**	***p*-value**	***p*-adj value**
CCL26	3.97728	15.75	0.000212	0.0126

Table S2. List of intratumoral DEGs from mice with Renca-Luc tumors (eKET vs Control)

**Downregulated**			
**Gene**	log2FoldChange	**Fold Change**	***p*-value**
Wdr76	-0.694869858	-1.61874	0.0074
Hif1a	-0.703879331	-1.62888	0.00859
Adm	-1.130060541	-2.18868	0.01948
Il18r1	-1.057496857	-2.08132	0.02311
Fanca	-0.739104505	-1.66914	0.04614
**Upregulated**			
**Gene**	**Log2 fold change**	**Fold Change**	***p*-value**
Sgk1	0.587192767	1.50232	0.01669
Tgfbr2	0.652010086	1.57136	0.03556
H2-t23	0.677937805	1.59985	0.04446
Ppl	0.894968784	1.85957	0.03312
Apoe	0.953882713	1.93708	0.00564
Ccnd1	1.115053827	2.16603	0.01012
Fcgr4	1.119555385	2.17281	0.03859
Lamc2	1.199211444	2.29614	0.03114
Wnt7b	1.289304828	2.44409	0.0385
Il2ra	1.435386145	2.70455	0.03462
Nt5e	1.629166573	3.09335	0.04054
Inhba	1.66789588	3.17752	0.02023
Mmp13	1.708291648	3.26773	0.04327
Itga2	2.017774702	4.04957	0.01496
Ptgs2	2.444518368	5.44345	0.00826
Tslp	2.710493383	6.54544	0.0096
Cpa3	2.970616193	7.83873	0.00854
